# PRODIG (Prevention of new onset diabetes after transplantation by a short term treatment of Vildagliptin in the early renal post-transplant period) study: study protocol for a randomized controlled study

**DOI:** 10.1186/s13063-019-3392-6

**Published:** 2019-06-21

**Authors:** E. Gaiffe, T. Crepin, J. Bamoulid, C. Courivaud, M. Büchler, E. Cassuto, L. Albano, J. M. Chemouny, G. Choukroun, M. Hazzan, L. Kessler, C. Legendre, Y. Le Meur, N. Ouali, A. Thierry, A. Anota, V. Nerich, S. Limat, F. Bonnetain, D. Vernerey, D. Ducloux

**Affiliations:** 1 CHU Besançon, Department of Nephrology, Dialysis, and Renal Transplantation, Federation Hospitalo-Universitaire INCREASE, F-25000 Besançon, France; 2 INSERM, UMR1098, EFS-BFC, University Burgundy Franche-Comte, LabEx LipSTIC, FHU INCREASE, F-25000 Besançon, France; 3 CHU Besançon, CIC Biothérapie, INSERM CIC1431, F-25000 Besançon, France; 4 CHU Bretonneau, Department of Nephrology and Clinical Immunology, EA 4245 Transplantation, Immunology, Inflammation, F-37044 Tours, France; 5 Pasteur hospital, L’Archet hospital group, Department of Nephrology, Dialysis, and Renal Transplantation, F-06000 Nice, France; 6 CHU de Rennes, Department of Nephrology, F-35033 Rennes, France; 7 CHU Amiens, Department of Nephrology, Dialysis, and Renal Transplantation, F-80054 Amiens, France; 8 CHU de Lille, Nephrology department, University of Lille UMR 995, F-59000 Lille, France; 9 CHU Strasbourg, Department of Endocrinology, Diabetes and Nutrition, F-67000 Strasbourg, France; 10 Necker hospital, Department of Nephrology, Dialysis, and Renal Transplantation, F-75743 Paris, France; 11 Department of Nephrology, CHU de Brest, UMR1227, Université de Brest, Inserm, F-29609 Brest, France; 12 Tenon hospital, Nephrological Emergencies and Kidney Transplantation, F-75571 Paris, France; 13 CHU de Poitiers, Department of Nephrology, Dialysis, and Renal Transplantation, F-86021 Poitiers, France; 14 CHU Besançon, Methodology and quality of life unit, F-25000 Besançon, France; 15 CHU Besançon, department of Pharmacy, F-25030 Besançon, France; 16 Service de Néphrologie et transplantation rénale, Centre Hospitalier Régional Universitaire de Besançon, 3, boulevard Alexandre Fleming, 25030 Besançon, cedex France

**Keywords:** Diabetes prevention, Vildagliptin, Kidney transplantation, Randomized controlled trial

## Abstract

**Background:**

Post-transplant diabetes is a frequent and serious complication of kidney transplantation. There is currently no treatment to prevent or delay the disease. Nevertheless, identification of risk factors make it possible to target a population at risk of developing de novo diabetes. We hypothesized that a short-term treatment with vildagliptin may prevent new onset diabetes after transplantation (NODAT) in high-risk patients.

**Methods/design:**

This is a multicenter, double-blind, placebo-controlled randomized clinical trial. Patients undergoing first kidney transplantation will be included from ten French transplant centers. Included patients will be randomized (1:1) to receive either vildagliptin 100 or 50 mg/day (depending on glomerular filtration rate) during 2 months (the first dose being administered before entering the operating theatres) or placebo. Additional antidiabetic therapy could be administered according to glycemic control. The primary outcome is the proportion of diabetic patients 1 year after transplantation, defined as patients receiving a diabetic treatment, or having a fasting glucose above 7 mmol/l, and/or with an abnormal oral glucose tolerance test. Secondary outcomes include glycated hemoglobin, the occurrence of acute rejection, infection, graft loss and patient death at 3 months, 6 months, and 12 months after transplantation. Outcomes will be correlated to clinical and general characteristics of the patient, cardiovascular history, nephropathy, dialysis history, transplantation data, biological data, health-related quality of life, and the cost-effectiveness of prevention of diabetes with vildagliptin.

**Discussion:**

We have scarce data on the pharmacological prevention of post-transplant diabetes. If our hypothesis is verified, our results will have a direct application in clinical practice and could limit diabetes-associated morbidity, reduce cardiovascular complications, increase quality of life of renal transplant patients, and consequently promote graft and patient survival. Our results may possibly serve for non-transplant patients carrying a high-risk of diabetes associated with other co-morbidities.

**Trial registration:**

ClinicalTrials.gov, NCT02849899. Registered on 8 February 2016.

**Electronic supplementary material:**

The online version of this article (10.1186/s13063-019-3392-6) contains supplementary material, which is available to authorized users.

## Background

New onset diabetes after transplantation (NODAT) affects 15 to 20% of renal transplant patients and is associated with increased morbidity and reduced survival of both transplants and patients [1, 2]. Furthermore, our group identified NODAT as a risk factor for atherosclerotic events [3]. The majority of NODAT occurs in the first months following transplantation. Corticosteroids, anti-calcineurin, and mammalian target of rapamycin (mTOR) inhibitors have a major diabetogenic impact and greatly contribute to the increase in diabetes prevalence after transplantation. Some pre-transplant risk factors have been identified, offering the opportunity to define an individual risk of NODAT. Considering its impact on graft and patient survival, the ability to predict NODAT is a major issue. Chakkera et al. [4, 5] reported that a simple equation using six easily available parameters provided a good discrimination of risk and a good identification of high-risk populations for targeted prevention. The capacity to predict NODAT occurrence is the prerequisite to efficiently prevent or delay the onset of diabetes using appropriate therapeutic interventions.

To date, few studies have been performed on the pharmacological prevention of NODAT. Hecking et al. [6] recently reported that a short treatment with insulin, administered immediately after transplantation, reduced the incidence of de novo diabetes one-year post-transplant. The occurrence of diabetes, a secondary endpoint, was reduced by 73% in the treated group. However, NODAT was a secondary endpoint and the side effects of insulin are not necessarily compatible with a preventive strategy. Relevant experimental evidence suggests that gliptins could be used in the pharmacological prevention of NODAT. Indeed, beyond the effects on blood glucose, gliptins have other actions, including a protective effect on β cells and anti-inflammatory action that could prevent or delay the occurrence of new-onset diabetes after renal transplantation in patients at risk [7–10]. The gliptins are approved for the treatment of type 2 diabetes, among which vildagliptin was used to treat diabetes after kidney transplantation in two clinical studies [11, 12]. These studies showed the anti-diabetic effect of vildagliptin taken early in the disease, but not as a preventive treatment for diabetes. Our hypothesis is that a short-term treatment of vildagliptin administered in the early post-transplant period may prevent the occurrence of NODAT.

## Methods/design

### Study design

This is a multicenter, double-blind, placebo-controlled randomized clinical trial. The study will include 186 patients receiving a kidney transplant and matching the criteria for inclusion. Recruitment will be done in ten French transplant units: Amiens, Besançon, Brest, Lille, Nice, Paris (Necker and Tenon), Rennes, Poitiers, and Tours. Patients will be randomized into two groups: group 1 will be treated with vildagliptin and group 2 will be treated with placebo. The treatment duration will be 2 months. The dose will be adjusted during treatment, depending on the results of glomerular filtration rate. The administration of vildagliptin begins before entering the operating theatre. Blood glucose is controlled and if necessary hyperglycemia will be treated according to current recommendations, regardless of the group and the study.

### Participants

#### Inclusion criteria

Graft recipients aged 18 or above will be eligible for inclusion if they:

- Receive a first kidney transplant
- Are considered at high risk of developing NODAT, having at least two of the three following criteria: age > 50 years; body mass index (BMI) > 30 kg/m^2^; direct family history of type 2 diabetes
- Can receive immunosuppressive therapy including tacrolimus, mycophenolic acid, and steroids
- Are patients in whom the cessation of steroids may be considered at the latest 3 months post-transplant
- Sign the informed consent for study participation
- Are affiliated with a medical care system

#### Non-inclusion criteria

Graft recipients will not be eligible for inclusion if they have the following conditions:

- Legal disability or limited legal capacity
- Patient unlikely to cooperate in the study and/or low early cooperation by the investigator
- Patient without health insurance
- Pregnancy and breast feeding
- Hepatic insufficiency
- Patient with class IV New York Heart Association (NYHA) heart failure
- Patient with galactose intolerance, Lapp lactase deficiency, or glucose-galactose malabsorption syndrome
- Patient in the period of exclusion of another study or under the “National Register of Volunteers”
- Inability to understand the reasons for the study; psychiatric disorders judged by the investigator to be incompatible with the inclusion in the study
- Active infection
- Infection with hepatitis C virus
- A history of diabetes
- A history of pancreatitis
- A history of angioedema
- Multi-organ transplantation

#### Participant recruitment

The study will be proposed to patients receiving a kidney transplant and matching the criteria of inclusion. The information sheet is given after admission for kidney transplant. The history of diabetes is checked during the inclusion consultation and in the medical file. Then the physician collects informed consent before going to the operating theatre. Recruitment will be done in ten French transplant units (Amiens, Besançon, Brest, Lille, Nice, Paris (Necker and Tenon), Rennes, Poitiers, and Tours). Delay may be shorter than the recommended time, but it corresponds to the studies on transplantation for which only a few hours are available between patient admission and surgery. Then, patients will be randomized.

#### Randomization

Patients will be randomized 1:1 into two groups using a minimization technique with stratification according to center and the number of risk factors for developing diabetes (two or three).

- Group 1 (interventional group) will be treated with vildagliptin 100 or 50 mg/day for 2 months (depending on whether their glomerular filtration rate is above or below 50 ml/min; Modification of Diet in Renal Disease (MDRD) equation).
- Group 2 (placebo group) will be treated with placebo according to the same dosage.

Random allocation sequence is generated using CleanWeb™. The minimization algorithm takes into account already randomized patients in order to allocate a new treatment to minimize difference between stratification criteria. The randomization result provided by the system is attributed in 80% of cases; otherwise, the other treatment is attributed. Neither the nurse nor the doctor nor the patient will know the nature of the ingested tablets. The blinding is created by the clinical research associate coordinator. The randomization number is assigned directly to the centers via CleanWeb™.

### Interventions

#### Timing of administration of the study treatment

First administration of the study treatment (vildagliptin or placebo) will be performed within hours prior to patient departure to the operating room. The dosage of vildagliptin will conform to the marketing authorization of data in its indication in the treatment of diabetes in kidney transplant patients. Patients will receive a half dose of therapy until they have a glomerular filtration rate greater than 50 ml/min of (MDRD equation). Patients will receive one oral tablet in the morning and one in the evening (100 mg per day) or just one in the morning (50 mg per day) for 2 months.

#### Posology change

The glomerular filtration rate will be measured each week and treatment changed accordingly. Blood glucose will be controlled and, if necessary, hyperglycemia will be treated according to current recommendations, regardless of the group and the study.

#### Premature discontinuation of the study treatment

All suspected adverse reactions related to the investigational medicinal product which occur during the present trial and that are both unexpected and serious are subject to expedited reporting. Based on the risk management plan for vildagliptin, subsequent adverse events will be followed attentively and considered as adverse events of special interest, including gastrointestinal disorders, pancreatitis, hepatitis, skin reaction, infection, neurotoxicity, pancreatic cancer, and cardiac disorders. These reactions will be recorded in the electronic case report form (e-CRF) and transmitted to the sponsor in cases of serious adverse events. In this case, the investigator reserves the right to prematurely terminate the study treatment for medical reasons. The reasons for premature discontinuation should be properly documented.

#### Appropriate administration of treatment units

A record of the number of tablets dispensed to and taken by each subject must be maintained and registered in the CRF. Compliance with therapy will be assessed through querying the patient during visits and examination of pillboxes given back at the endpoint.

#### Unblinding modalities

If serious adverse events attributable to the study product occur, a blind lift procedure may optionally be initiated both by investigators and by the pharmacovigilance unit of the University Hospital. In the latter case, the unblinding will be carried out by the data manager on request of the head of pharmacovigilance. Clinicians as well as biostatisticians retain the blind.

#### Concomitant medication

Vildagliptin is an approved treatment for NODAT in patients with renal failure. It will be used in these patients as a preventive treatment according to the same recommendations as in curative treatment. Vildagliptin has a low potential for drug interactions. It is neither a substrate nor an inhibitor or inducer of cytochrome P450 (CYP450) enzymes; therefore, it is unlikely to interact with active substances that are substrates, inhibitors, or inducers of these enzymes. Clinical studies showed no pharmacokinetic interactions clinically relevant to vildagliptin with other antidiabetic treatments (pioglitazone, metformin and glibenclamide, digoxin, warfarin, amlodipine, ramipril, valsartan, or simvastatin). Therefore, no treatment (antidiabetic or not) is prohibited because of the study. As with other oral antidiabetic agents, the hypoglycemic effect of vildagliptin may be reduced by certain active substances, including thiazides, corticosteroids, thyroid hormones, and sympathomimetics. As in conventional treatment, however, these treatments are not prohibited. In conclusion, the prescription of vildagliptin for this study does not modify medical care of included patients (whether for immunosuppressive treatment or for antidiabetic treatments).

### Study outcomes

The primary endpoint is the proportion of diabetic patients 1 year after transplantation, defined as one of the following criteria: patients receiving a diabetic treatment, patients have a fasting glucose above 7 mmol/l, and/or patients with an abnormal oral glucose tolerance test (OGTT).

The criteria for secondary assessments are: glycated hemoglobin (HbA1c) 3 months, 6 months, and 12 months after transplantation; occurrences of acute rejection, infection, graft loss, and patient death 3, 6, and 12 months after transplantation; clinical history (cardiovascular history, baseline renal disease, dialysis, anti-human leukocyte antigen antibodies, dyslipidemia, hypertension, type of donor, duration of ischemia, cytomegalovirus (CMV) status, CMV prophylaxis, pneumocystis prophylaxis); biological data (creatinine, uric acid, C reactive protein (CRP), blood sugar balance, lipid profile, liver function tests, pancreatic balance, and blood counts); health-related quality of life (HRQoL; Renal Transplant Quality of Life (ReTransQoL) questionnaire), 8 days and 3, 6, and 12 months after transplantation; and the cost-effectiveness of prevention of diabetes with vildagliptin correlated to outcomes.

### Study visits

**Fig. 1:**
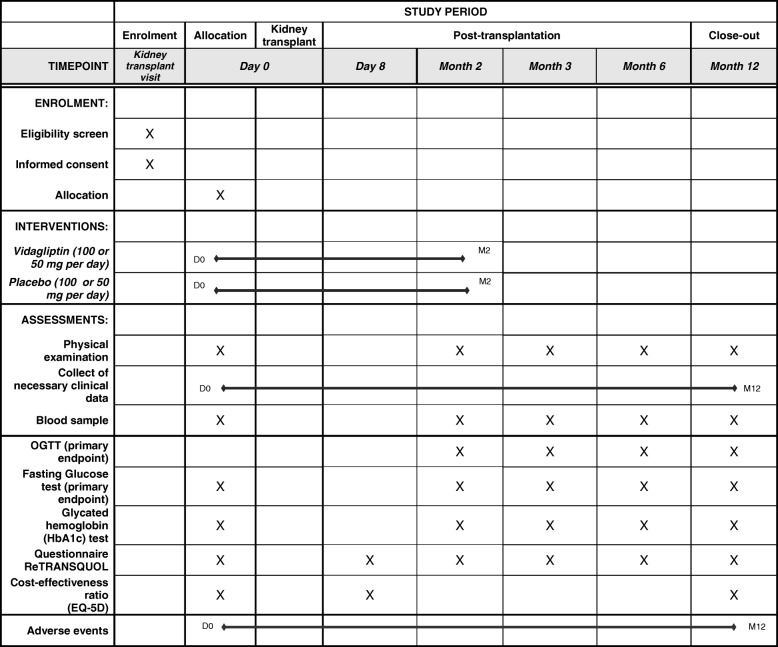
SPIRIT figure of the PRODIG study

As presented in Fig. 1 (Additional file 1), the study will include five visits (D0, D8, M3, M6, and M12) corresponding to those taking place in the context of the current follow-up after a kidney transplant. During these visits, clinical examination and a blood test will be conducted as part of the study to collect necessary clinical data in each center on case report forms and to determine the different dosages in the hospital laboratories, respectively. Blood glucose will be controlled and, if necessary, hyperglycemia will be treated according to current recommendations, regardless of the group and the study. The HRQoL will be estimated using the ReTransQoL questionnaire and the health-utility with the EQ-5D questionnaire (EuroQol questionnaire with five dimensions). The benefits of prevention of NODAT will be evaluated by estimating the cost of patient care during the first year of transplantation (care, medicines, medical examinations, hospitalization, etc.).

### Adverse effects

The risks associated with participation in the study are those related to an additional blood sample, either hematoma, redness, vagal malaise, and those related to the glucose tolerance test, including nausea, vomiting, malaise, sweating, tremors, palpitations, hunger, and confusion. The risks associated with taking vildagliptin are those described for its use as an antidiabetic treatment (vildagliptin ((S)-1-[N-(3-hydroxy-1-adamantyl)glycyl]pyrrolidin-2-carbonitrile; N° Chemical Abstracts Service, 274901165). Based on the risk management plan for vildagliptin, adverse events will be following attentively and considered as adverse events of special interest, including gastrointestinal disorders, pancreatitis, hepatitis, skin reaction, infection, neurotoxicity, pancreatic cancer, and cardiac disorders. Particular attention will be given to adverse events occurring at the national level, including hepatitis, pancreatitis, rash, infections, pancreatic cancer, bullous eruption, and cardiovascular serious adverse events. In addition, when combined, inhibitors of angiotensin converting enzyme and antagonists of angiotensin II receptor (ARA2) will be used with caution.

### Statistical analysis plan

#### Sample size and power calculation

The estimated proportion of diabetic patients 1 year after transplantation is 40% in group 2 (P_2_) and the expected decrease in group 1 (P_1_) is 50% (i.e., 20% at 1 year) [4, 5]. The expected 50% decrease is estimated according to the results obtained with the insulin treatment of Hecking et al. (100% decrease at 1 year) [6]. With the hypotheses H0 (P_2_ − P_1_ = 0; null hypothesis) and H1 (P_2_ − P_1_ ≠ 0; altenative hypothesis) and by considering a group sequential design with a Z score test (pooled estimate for variance estimation), a bilateral alpha risk of 5% and a ß risk of 20%, it will be necessary to randomize 84 patients per group (a total of 168 patients). We expect 10% of patients at 1 year to be non-evaluable and thus we will include 186 patients in total. An interim analysis is planned by the statistician of the investigation team (without the investor being aware of the lifting of the blind) to reject H0 or H1 when at least 84 patients are randomized with one year of follow-up (42 in each group; 50% of the information fraction) using alpha spending function and O’Brien Fleming boundaries. Sample size calculations were performed with the EAST 6 software.

Moreover, as supportive analysis with this sample size will be able to demonstrate an improvement in the time of diabetes-free occurrence from 40% to 20% (hazard ratio of 0.43) with a bilateral alpha risk of 5% and we will have 90% statistical power with 63 events.

### Statistical analysis

The statistical analysis plan (final and HRQoL analyses) will be written and approved before the database is frozen.

#### Primary outcome analysis

The intention to treat (ITT) population will be used for efficacy parameter analysis (all randomized patients whatever the eligibility criteria and treatment received).

A Z score test with pooled estimate for variance estimation will be used to compare the estimated proportion of diabetic patients 1 year after transplantation between the two arms. A Lan-DeMets alpha spending function with O’Brien and Fleming boundaries (function depending on the information fraction in the ITT population) will be used. For the interim analysis with 84 informative patients at 1 year of follow-up (50% of the information fraction) we will reject H0 (efficacy) or reject H1 (futility) if the test p value is lower than or equal to 0.0003 and higher than 0.715, respectively. If the p value is higher than 0.0003 but lower than or equal to 0.715, we can’t make a conclusion and the study will continue. At the end of the study with 168 informative patients at 1 year of follow-up, we will reject H0 and then accept H1only if the test p value is lower than or equal to 0.049.

#### Secondary outcome, exploratory, and sensitivity analyses

Selected efficacy analyses will be repeated in the per protocol population (i.e., subset of the ITT population meeting the following criteria: all eligibility criteria fulfilled, at least one dose of allocated treatment administered). The safety population will be used for reporting the safety and treatment exposure data. A modified ITT population will be used for HRQoL analysis, considering all randomized patients with at least the baseline HRQoL questionnaire available. Continuous variables will be summarized using descriptive statistics, i.e., the number of patients with available data (n), mean, median, standard deviation (SD), 25–75% quartile (Q1–Q3), minimum, and maximum. Continuous variables could be transformed to categorical variables using the median or using conventional cut-offs from bibliography or clinical practice. Frequencies in tables will be presented by arm, total frequency, percentages, and missing modality. Qualitative variables will be summarized by means of counts and percentages. Unless otherwise stated, the calculation of proportions will be based on the sample size of the population of interest. The rate of incidence of NODAT will be determined using confidence interval (CI) and compared using Chi 2 test. Univariate and multivariate logistic analyses will be done to identify the independent prognostic factor of the rate of incidence of NODAT and to compute the odds ratio with 95% CI. The continuous variables will be treated as quantitative and qualitative data using cut-offs in anticipation of elaborating a practical clinical tool. Factors will be considered for inclusion in the model if the univariate p value is ≤ 0.1. Interactions between treatment and some variables will be tested at a two-sided 10% level (i.e., a p value > 0.1 indicates no evidence of heterogeneity of treatment effect across the subgroups for each factor). All tests will be performed in an exploratory purpose at a two-sided 5% significance level.

##### Health-related quality of life

The minimal clinically important difference will be fixed to 10 points for each HRQoL score. A descriptive analysis of the scores obtained for each questionnaire at baseline and at each follow-up will be performed using n, mean (SD), and median (range) for all patients and according to the treatment arm. A linear mixed model for repeated measure will be applied to HRQoL scores integrating all measurement times, including a treatment effect, a time effect, and an interaction between time and treatment. The time to HRQoL score deterioration (TTD) approach will also be explored. The TTD will be defined as the time from inclusion in the study to the first deterioration of at least 10 points of the HRQoL score compared to the baseline score [13, 14]. The TTD will be determined according to the Kaplan-Meier estimation method and compared by treatment arm using the Log-rank test. Univariate and multivariate Cox regression models will be performed in order to estimate hazard ratio with 95% CI to investigate potential factors independently associated with the TDD.

All tests in HRQoL analyses will be performed at a two-sided 1% significance level to prevent inflation of the alpha type one error with a Bonferroni approach for the five targeted dimensions (5%/5 = 1%; physical health, mental health, medical care and satisfaction, treatment, and fear of losing the graft).

The EQ-5D questionnaire is a standardized tool consisting of five dimensions: mobility, self-care, usual activity, pain and discomfort, and anxiety and/or depression. Each patient’s quality-adjusted life year will be calculated, from D0 until M12 or censoring date, by multiplying the duration of each period by the corresponding period’s utility score.

##### Pharmacoeconomic impact

The total number of NODAT in the early post-transplant period avoided and the total number of acute rejections avoided will be used as the measure of effectiveness. Because effectiveness and direct medical costs will be measured over a period of one year, discounting will not apply to clinical and economic parameters according to recommendations of the Department of Economics and Public Health Assessment of the French Haute Autorité de Santé [15]. The incremental cost-effectiveness ratio will be calculated comparing both interventions using the formula: (Cost group 1 − Cost group 2)/(Effectiveness group 1 − Effectiveness group 2). The robustness of the cost-effectiveness analysis (CEA) will be assessed through only deterministic sensitivity analyses. Different one-way univariate, deterministic sensitivity analyses will performed for parameters likely to influence the results of the CEA.

### Ethical issues

#### Ethics committee approval

The trial has been approved nationally by the Advisory Committee on Information Processing for Research in the Field of Health (N°MEDAECNAT-2018-03-00009, N°EudraCT 2016–002023-28) and the Committee for Personal Protection (National approval by CPP Ile de France XI, n°17,039; Additional files 3 and 4).

#### Information and consent forms

All participants will be informed to the fullest extent possible about the study, in a language and terms they are able to understand. Prior to a subject’s participation in the clinical study, the written informed consent form should be signed, name filled in, and personally dated by the subject or by the subject’s legally acceptable representative, and by the person who conducted the informed consent discussion. A copy of the signed and dated written informed consent form will be provided to the subject.

### Data quality and regulatory issues

#### Monitoring of the study

The sponsor of this clinical study, the Besançon University Hospital, is responsible to health authorities for taking all reasonable steps to ensure the proper conduct of the clinical study as regards ethics, clinical study protocol compliance, and integrity and validity of the data recorded on the case report forms. At regular intervals during the clinical study (every five or six patients for each center or at least every year) a representative of the monitoring team will review study progress, investigator and subject compliance with clinical study protocol requirements, and any emergent problems. These monitoring visits will include but not be limited to review of the following aspects: subject informed consent, subject recruitment and follow-up, serious adverse event documentation and reporting, adverse event documentation and reporting, and source document requirements. According to the ICH guidelines for Good Clinical Practice, the monitoring team must check the case report form entries against the source documents, except for the pre-identified source data directly recorded in the case report form.

#### Data Management of the study

Clinical data management is performed at the Methodology and quality of life unit in oncology of University Besançon hospital using SAS version 9.4 (SAS Institute, Cary, NC, USA). Originals of all study-related report forms will be stored in the study headquarters at the trial site (according to the respective national laws). The data will be provided as SAS® files to the statistical team for data analysis using SAS version 9.4 and R software version 2.15.2 (R Development Core Team, Vienna, Austria; https://www.r-project.org/).

Investigators store all administrative documents, patient identification logs, signed patient consent forms, copies of the data documentation forms, and common study documentation. Original data for study patients (medical records) will be stored. A list allowing patient identification will be kept for 15 years (directive 2001/83/EG). The Investigator should retain the study documents at least 15 years after the completion or discontinuation of the clinical study.

## Discussion

### Conditions for patient selection

Patients considered at high risk of developing NODAT are included in the study. The selection of these patients is based on the Chakkera et al. criteria [4, 5]. They reported that an equation using six parameters provides good identification of high-risk populations for targeted prevention. These parameters include age, planned corticosteroid therapy post-transplant, prescription for gout medicine, BMI, fasting glucose and triglycerides, and family history of type 2 diabetes. The two parameters planned corticosteroid therapy post-transplant and prescription for gout medicine were not retained because they included a significant bias due to the subjectivity of clinician prescription. In addition, the determination of fasting glucose and triglycerides cannot be selection criteria because they both require fasting for 12 h, which is not possible for patients selected for a kidney transplant. Nevertheless, the discriminating power of a simpler algorithm is also well established. The risk of developing NODAT is multiplied by 20 in patients over 55 years of age with a BMI greater than 30 kg/m^2^ (unpublished data from [16]). Accordingly, three criteria have been retained: age over 50 years, BMI greater than 30 kg/m^2^, and a direct family history of type 2 diabetes.

### Justification of the choice of vildagliptin, dosing and timing of drug administration

Hecking et al.’s previous study [6] reported that insulin, administered immediately after transplantation, was an efficient pharmacological prevention of NODAT. This study included 50 renal transplant patients and showed that a 3-month treatment of neutral protamine Hagedorn insulin decreased HbA1c and the occurrence of diabetes (73%) in the treated group. However, NODAT was a secondary endpoint and hypoglycemia occurred five times in the treatment group [6]. This strategy seems incompatible with preventive treatment. Moreover, the low numbers of patients and the absence of double blinding hamper the conclusions of the study.

Other non-hypoglycemic antidiabetic drugs seem to be better candidates for the prevention of diabetes. Among them, gliptins seem to be the appropriate therapy because of their mechanistic properties. These drugs are inhibitors of dipeptyl peptidase-4 (DPP-4), which inactivates the incretins, the glucagon-like peptide-1 (GLP-1), and the gastric inhibitory polypeptide (GIP). DPP-4 inhibition causes an increase in the GLP-1 and GIP concentrations which induce insulin secretion and inhibition of glucagon secretion. Beyond the effects on blood glucose, gliptins have pleiotropic effects, including:

- A protective effect on β cells. Many preclinical studies have demonstrated the anti-proliferative and apoptotic effects of gliptins on β cells [7]. The diabetogenic effects of tacrolimus are the consequence of increased insulin resistance, but also a direct effect on β cells. Tacrolimus induces apoptosis in β cells, decreases insulin exocytosis, and inhibits transcription of the insulin gene [8]. Gliptins could prevent these deleterious effects.
- Anti-inflammatory effects. DPP-4 is strongly expressed on many immune cells, including monocytes and T lymphocytes. Inhibition of DPP-4 reduces the production of various cytokines, including interleukin (IL)-2, IL-12, and interferon-γ. Transforming growth factor-β is up-regulated after inhibition of DPP-4. Inhibition of DPP-4 also exerts effects on chemokines [9]. It follows from these properties that gliptins have anti-inflammatory action. Inflammation plays an important role in the onset of NODAT [10]. These different effects of gliptins could prevent or delay the occurrence of NODAT in patients at risk. Several members of the gliptins can be used in the treatment of diabetes; sitagliptin and vildagliptin are the best candidates. However, there is a significant risk of acute pancreatitis with sitagliptin therapy, and sitagliptin should not be used in patients with moderate to severe renal impairment [17]. Vildagliptin was used to reduce diabetes after kidney transplantation in two clinical studies. Haidinger et al. [11] conducted a randomized, double-blind, phase II trial to assess the efficiency of the administration of 50 mg of vildagliptin per day versus placebo for 3 months to 32 patients (16 per group) transplanted for at least 6 months and newly diagnosed for diabetes by NODAT score. Treatment with vildagliptin significantly improved oral glucose tolerance and reduced the glycated hemoglobin (p < 0.01) but did not affect fasting glucose. The differences were not maintained one month after treatment. Werzowa et al. [12] conducted a similar study comparing vildagliptin and pioglitazone versus placebo. Forty-eight patients (16 per group) were recruited using the same method as above and followed until the end of treatment, namely 3 months. As in the study of Haidinger et al., treatment with vildagliptin improved oral glucose tolerance and reduced glycated hemoglobin but did not alter fasting blood glucose. Given the important effect of vildagliptin taken early in diabetes, we can reasonably expect a preventive effect. Vildagliptin is prescribed according to the dosage indicated by the High Health Authorities for use in NODAT.

### Justification of the primary outcome

The primary endpoint is the proportion of diabetic patients 1 year after transplantation, defined as patients receiving a diabetic treatment, patients with a fasting glucose above 7 mmol/l, and/or patients with an abnormal OGTT. Unlike the Hecking et al. [6] study, we chose de novo diabetes, a clinically relevant criterion, as the main criterion and not a surrogate marker. This will actually determine if this preventative treatment effectively protects from the disease. Consequently, the results will be expressed as the number of patients with diabetes, as defined previously, at a given time. At transplant, 100% of patients will be considered as diabetic because all patients will be treated, at least, with vildagliptin (or placebo) for two months. At 2 months, treatment with vildagliptin, prescribed in the context of the study, will be stopped and only some patients will be considered diabetic (abnormal dosages and/or prescription of a new antidiabetic treatment) at 3 months. This number of patients may still vary at 6 months and one year post-transplant [6]. The main criterion is the number of patients with diabetes at one year.

The onset of diabetes at other times (3 and 6 months) as well as abnormal glucose indicators (fasting glucose, OGTT, glycated hemoglobin) at each time will be assessed as secondary endpoints. These data will also be correlated with the clinical events (occurrence of acute rejection, infection, graft loss, and patient death at 3, 6, and 12 months after transplantation), the medical history, and the biological assessment of the patients.

### Health-related quality of life, renal transplantation, and diabetes

The HRQoL assessment significantly predicts patient survival, particularly in patients with chronic kidney disease [18, 19]. Moreover, as transplantation results in one of the best increases of patients’ quality of life, its estimate is essential in the treatment evaluation of this population [15, 20–22]. It has been shown that cardiovascular complications, dialysis method, hypertension, method of diabetes treatments (oral or injectable), and hypoglycemia influence the quality of life [19, 23, 24]. Furthermore, in patients with type II diabetes, hypoglycemia is associated with depressive symptoms and heightened anxiety and other factors important for quality of life [25]. Prospective reports have indicated that diminished HRQoL among adults with type II diabetes is associated with subsequent mortality risk [26]. Kizilisik et al. [27] have demonstrated in a cohort of 86 kidney transplant patients that transplantation and diabetes have direct, opposite, and independent effects on the quality of life of patients.

Although the most common questionnaire used for assessing HRQoL is the Short Form 36 (SF-36), Gentile et al. [28] have developed a quality of life questionnaire to specifically assess the quality of life associated with renal transplantation in France. This questionnaire was analyzed, evaluated, and validated by French public health priorities [29]. That is why the HRQoL questionnaire ReTransQoL will be used in our study [28, 30]. The ReTransQOL questionnaire was completed at t0, 8 days, 3 months, 6 months, and 12 months after transplantation. The questionnaire is done on day 0 and day 8 for two reasons: to get rid of the beneficial effect on patient’s quality of life due to transplant announcement and to overcome the impossibility of filling out the form just before entering the operative block. The EQ-5D questionnaire allows evaluation of a wide variety of interventions. It is widely used in utility calculations. It has evidence of validation in type 2 diabetes with a variety of different profiles (treatment, symptoms, and complications) [31].

### Health economics analysis

The additional cost associated with NODAT could be significantly reduced by efficient prevention. A USA study found that, for the period between 1994 and 1998, a newly diagnosed diabetic patient cost $21,500 in medical expenses 2 years after transplantation [32]. The increase in cardiovascular diseases due to post-transplantation diabetes may account for this additional cost.

The purpose of this health economics analysis will be to investigate the cost-effectiveness of prevention of diabetes with vildagliptin in patients included in the PRODIG clinical trial. The CEA will be performed on resources and effectiveness collected prospectively and aggregated from all patients included in the PRODIG clinical trial. The analysis will be performed from a health care payer perspective. Only direct medical costs will be computed from the inclusion visit of a patient (D0) until the last visit (M12) (i.e. 1-year-long time horizon) [15]. They will include medication (as preventive treatment of diabetes and kidney transplant rejection, lipid-lowering therapy, diabetes treatment, treatment of diabetes and renal transplant complications, treatment of adverse event), hospitalization (complications related to the kidney transplant and/or new diabetes, serious adverse event management, follow-up), inpatient and outpatient consultations, biomedical analyses (glomerular filtration rate, OGTT, Hba1c, proteinuria, serology for human immunodeficiency virus, hepatitis B and C viruses, CMV), and transport. Minor costs and costs considered to be independent of the treatment arm will not be taken into account; nor will indirect medical and intangible costs. Costs will be expressed in Euros (€; reference year 2018). Each cost will be calculated using the official tariff, as, for example, for each hospitalization, the national health-insurance provider’s tariffs for Diagnosis-Related Group.

Furthermore, health utility-based measures of quality of life are recommended by some decision makers in health care. No studies have addressed changes in utility in patients with the same profile as patients included in the PRODIG clinical trial. The purpose of this secondary analysis will be to assess the association of change in health utility with changes in other variables such as complications or treatment. Some preference-based measures of health status have been developed to facilitate the use of utilities in economic analyses. The EQ-5D is one of the most widely used descriptive systems for health state, recommended by the National Institute for Health Care and Excellence in the UK for the incorporation of health benefits in cost-utility analysis and for which validated preference-based scores are available in France [20–22, 30].

### Clinical implications

The incidence of type 2 diabetes is steadily increasing after solid organ transplantation [33]. In kidney transplantation 30% of patients develop diabetes within 6 months of transplantation. Controlling this impaired glucose metabolism is a challenge in transplantation. We have only few data on the pharmacological prevention of NODAT. In our study, we hope to show that the use of the antidiabetic vildagliptin will achieve similar results to the Hecking et al. study without the inconvenience of direct insulin treatment (subcutaneous administration, hypoglycemia). Indeed, in addition to the side effects of treatment (hypoglycemia, malaise, etc.), the use of insulin therapy is very controversial. Hecking et al. indicate in their study that the administered treatment results in a HbA1c rate that is too low (< 6%) [6], which correlates with increased cardiovascular complications [34]. If our hypothesis is verified, our results will have direct application in clinical practice. Moreover, direct benefit is expected for the patients in the experimental group. Indeed, vidalglipin administration should prevent the occurrence of NODAT and thus limit the associated morbidity, reduce cardiovascular complications, and consequently promote graft and patient survival. Prevention of NODAT would also increase the quality of life in renal transplant patients. Importantly, a double-blind study has never been accomplished in this area. Our results may serve for non-transplant patients carrying a high-risk of diabetes associated with other co-morbidities such as cardiovascular disease.

### Trial status

At the time of submission, the regulatory authorizations have been obtained (protocol version 6 dated May 22, 2018), seven patients have been enrolled in the study in our center (first inclusion 2018/10/26), and implementation is in progress in the participating hospitals (signing of conventions, implementation meeting). The recruitment will be completed in approximately November 2020.

## Additional files

Additional file 1: SPIRIT 2013 checklist: recommended items to address in a clinical trial protocol and related documents. (PDF 82 kb)

Additional file 2: Financing: Proof of financing from the French Ministry of Health. (PDF 15 kb)

Additional file 3: Ethical approval document from the Committee for Personal Protection. (PDF 39 kb)

Additional file 4: Ethical approval document from Advisory Committee on Information Processing for Research in the Field of Health. (PDF 408 kb)

## Abbreviations

(e)CRF: (electronic) Case report form
(m)ITT: (modified) Intention-to-treat
ARA2: Antagonists of angiotensin II receptor
BMI: Body mass index
CEA: Cost-effectiveness analysis
CI: Confidence interval
CMV: Cytomegalovirus
CPP: Committee for Personal Protection
CRP: C reactive protein
CYP450: Cytochrome P450
DPP-4: DiPeptyl peptidase-4
EQ-5D: EuroQol questionnaire with five dimensions
GIP: Gastric inhibitory polypeptide
GLP-1: Glucagon-like peptide-1
HbA1c: Glycated hemoglobin
HRQoL: Health-related quality of life
ICH: International Council for Harmonisation
IL: Interleukin
ITT: Intention to treat
MDRD: Modification of diet in renal disease
mTOR: Mammalian target of rapamycin
NODAT: New onset diabetes after transplantation
NYHA: New York Heart Association
OGTT: Oral glucose tolerance test
ReTransQoL: Renal transplant quality of life
SD: Standard deviation
SF-36: Short Form-36
TDD: Time to definitive deterioration
