# Supplementary material for: PRODIG (Prevention of new onset diabetes after transplantation by a short term treatment of Vildagliptin in the early renal post-transplant period) study: study protocol for a randomized controlled study
Source: Trials. 2019 Jun 21;20:375. doi: 10.1186/s13063-019-3392-6 (PMC6588872; doi:10.1186/s13063-019-3392-6)
Supplement: Supplementary file 2 — Financing: Proof of financing from the French Ministry of Health. (PDF 15 kb) [file 13063_2019_3392_MOESM2_ESM.pdf]

Legal FINESS	Name of the health facility fund manager	Project leader / Investigator coordinator NAME	Project leader / Investigator coordinator FIRST NAME	Project title	Project number	Acronym	Escrow authorization (€)
250000015	CHU BESANCON	Gaiffe	Emilie	Prevention of new onset diabetes after transplantation by a short term treatment of Vildagliptin in the early renal post-transplant period	PHRC-15-384	Prodig	**335 395**
