# Supplementary material for: PRODIG (Prevention of new onset diabetes after transplantation by a short term treatment of Vildagliptin in the early renal post-transplant period) study: study protocol for a randomized controlled study
Source: Trials. 2019 Jun 21;20:375. doi: 10.1186/s13063-019-3392-6 (PMC6588872; doi:10.1186/s13063-019-3392-6)
Supplement: Supplementary file 3 — Ethical approval document from the Committee for Personal Protection. (PDF 39 kb) [file 13063_2019_3392_MOESM3_ESM.pdf]

CPP Ile de France XI
Committee of Personnal Protection

Saint-Germain-en-Laye, May 17, 2018

CHRU de BESANCON
Ingrid TISSOT
2 place St Jacques
25030 BESANCON CEDEX

Title of the essay:	**« Prévention du diabète de novo après transplantation par un traitement court par vildagliptine dans la période précoce suivant une transplantation rénale »**
Promotor:	**CHRU DE BESANCON**
Coordinator:	**Pr Didier DUCLOUX**
Promotor ref.:	**PRODIG**
IDRCB number :	**2016-002023-28**
Réf. CPP :	**17039**

Request for an opinion, valid authorization, on a category 1 search	**x**
Request for an opinion, valid authorization, on a category 2 search	
Request for an opinion, valid authorization, on a category 3 search	
Request for an advisory opinion on a substantial change of purpose in the use of a collection of elements human biological (art. L. 1211-2 of CSP)	
Request for an Advisory Opinion on a draft declaration of constitution of a collection of human biological samples (art. L. 1243-3 of CSP)	

Examinated documents	Version number and date
**Notice Mail**	2017/06/19
Additional document	November 20, 2017
Insurance	2017/06/19
Formulaire de demande	2017/06/19
Protocole	V4 of 2017/09/26
Summary	V1 of 2017/06/19
Summary of product characteristics	Attachment
Investigators' CV	Attachment
List of investigators	V2 of 2017/11/20
Information Form and Patient Informed Consent Form	V3 of 2018/04/19
Certificate of identity	2017/03/08
ReTRANSQOL and EQ-5D questionnaires	Attachment
Justification of the adequacy of the means	V1 of 2017/06/19

Michèle CATZ, présidente – **Sabine de la PORTE**, Vice-présidente – **Jean-François LAIGNEAU**, Secrétaire
Anne-Elisabeth DECARIS, Assistante
Pavillon Jacques Courtois – 2ème étage, 20, rue Armagis 78105 Saint Germain en Laye Cedex
Tél : 01.39.27.42.58 - Fax : 01.39.27.49.01
E.mail : cppidf11@chi-poissy-st-germain.fr

CPP Ile de France XI
Committee of Personnal Protection

	**PRODIG**
N° IDRCB :	**2016-002023-28**
Réf. CPP :	**17039**

I received the changes requested at the meeting of SEPTEMBER, 14 2017, concerning the research project referenced above.

Participated in the deliberation :

I – FIRST COLLEGE

**Annie DURAND**	**Hospital Pharmacist**
**Agnès GUIBERT**	**Biostatistician**
**Gérard LOEB**	**Doctor**
**Kolia MILOJEVIC**	**Biostatistician**
**Sabine de la PORTE**	**Searcher**

II - SECONDE COLLEGE

**Christine GHESTEM**	**Association of the families of Vésinet**
**Odile LACHAUD**	**Representative UDAF 78**
**Olivier LANTRES**	**Lawyer**
**Christine STOUFFLET**	**Philosopher**

No deliberative member of the committee is affected by a conflict of interest.

Therefore I give the FAVORABLE OPINION of our Committee for this research. Please believe, Madam, the assurance of my best feelings.

Michèle CATZ, chair of the meeting.

Michèle CATZ, présidente – **Sabine de la PORTE**, Vice-présidente – **Jean-François LAIGNEAU**, Secrétaire
Anne-Elisabeth DECARIS, Assistante
Pavillon Jacques Courtois – 2ème étage, 20, rue Armagis 78105 Saint Germain en Laye Cedex
Tél : 01.39.27.42.58 - Fax : 01.39.27.49.01
E.mail : cppidf11@chi-poissy-st-germain.fr
