# Supplementary material for: PRODIG (Prevention of new onset diabetes after transplantation by a short term treatment of Vildagliptin in the early renal post-transplant period) study: study protocol for a randomized controlled study
Source: Trials. 2019 Jun 21;20:375. doi: 10.1186/s13063-019-3392-6 (PMC6588872; doi:10.1186/s13063-019-3392-6)
Supplement: Supplementary file 4 — Ethical approval document from Advisory Committee on Information Processing for Research in the Field of Health. (PDF 408 kb) [file 13063_2019_3392_MOESM4_ESM.pdf]

ansm
Agence nationale de sécurité du médicament et des produits de santé

CLINICAL TRIAL AUTHORIZATION OF DRUG FOR HUMAN USE

Date : 04 JUIN 2018

Identifier of the clinical trial			
Title	PRODIG : Prevention of new onset diabetes after transplantation by a short term treatment of Vildagliptin in the early post-transplant period		
Promotor	CHU Besançon		
Ref. to remember	MEDAECNAT-2018-03-00009	N° EudraCT	**2016-002023-28**

Sender	Recipient (applicant: name / company / phone)	
ANSM / Direction Produit Médicaments en cardiologie, rhumatologie, stomatologie, endocrinologie, gynécologie, urologie, pneumologie, ORL, allergologie / Equipe ENDOC	Ingrid TISSOT<br>CHU Besançon<br>03 81 21 84 27	
Folder followed by Anne LAURENT<br>Tel : 33 (0) 1 55 87 32 80 / Fax : 33 (0) 1 55 87 30 53<br>Mail : anne.laurent@ansm.sante.fr	Mail	itissot@chu-besancon.fr

CPP recipient	Ile-de-France XI (Saint-Germain - Versailles)	Mail	cppidf11@chi-poissy-st-germain.fr

Given the public health code and in particular Article L. 1123-8, and the regulatory provisions adopted for its application, and having regard to the clinical trial authorization application file sent to the National Agency for the Safety of Medicines and health products (ANSM);

Considering the supplements filed by the promoter on May 28th, 2018 and in particular the protocol of the test cited in modified object (version 6 dated May 22, 2018) following the request of the ANSM;

The authorization mentioned in article L. 1123-8 of the Public Health Code is granted for the clinical trial mentioned above.

I ask you to send any request for modifications concerning this file by email addressed to the box: ams-essaiscliniques@ansm.sante.fr. When sending these files, I ask you to ensure that the subject of the message is marked: **MSA / EUDRACT No.** for SM submitted for authorization or for mixed dossiers (with modifications submitted for authorization and others for information).

Direction des médicaments en cardiologie, rhumatologie, stomatologie, endocrinologie, gynécologie, urologie, pneumologie, ORL, allergologie
Pôle « endocrinologie, gynécologie, urologie, pneumologie, ORL, allergologie »
Docteur Isabelle YOLDJIAN
Chef Produit

Confidentialité
Cette transmission est à l'attention exclusive du(des) destinataires ci-dessus mentionné(s) et peut contenir des informations privilégiées et/ou confidentielles. Si vous n'êtes pas le destinataire voulu ou une personne mandatée pour lui remettre cette transmission, vous avez reçu ce document par erreur et toute utilisation, révélation, copie ou communication de son contenu est interdite. Si vous avez reçu cette transmission par erreur, veuillez nous en informer par téléphone immédiatement et nous retourner le message original par courrier. Merci.

Confidentiality
This transmission is intended to the addressee(s) listed above only and may contain preferential or/and confidential information. If you are not the intended recipient, you are hereby notified that you have received the document by mistake and any use, disclosure, copying or communication of the content of this transmission is prohibited. If you have received this transmission by mistake, please call us immediately and return the original message by mail. Thank you.
